# Supplementary material for: Prevalence of Newly Diagnosed Malignancies in Patients with Polymyalgia Rheumatica and Giant Cell Arteritis, Comparison of 18F-FDG PET/CT Scan with Chest X-ray and Abdominal Ultrasound: Data from a 40 Week Prospective, Exploratory, Single Centre Study
Source: J Clin Med. 2020 Dec 4;9(12):3940. doi: 10.3390/jcm9123940 (PMC7762038; doi:10.3390/jcm9123940)
Supplement: Supplementary file 1 [file jcm-09-03940-s001.pdf]

Supplementary Table 1: Prevalence of cancer and MGUS in the included patients.

	Number of cancer patients (% (95% CI))				Number of MGUS patients (% (95% CI))			
Age groups	Male	Female	Total	P value[1]	Male	Female	Total	P value[1]
50 – 59	0/2 (0% (0 – 84.2%))	0/4 (0% (0 – 60.2%))	0/6 (0% (0 – 46.0%))	-	0/2 (0% (0 – 84.2%))	1/4 (25% (0.6 – 80.6%))	1/6 (16.7% (0.4 – 64.1%))	0.99
60 – 69	0/9 (0% (0 – 33.6%))	0/13 (0% (0 – 24.7%))	0/22 (0% (0 – 15.4%))	-	0/9 (0% (0 – 33.6%))	0/13 (0% (0 – 24.7%))	0/22 (0% (0 – 15.4%))	-
70 – 79	1/14 (7.1% (0.2 – 33.9%))[2]	1/24 (4.2% (0.1 – 21.1%))[3]	2/38 (5.3% (0.6 – 17.7%))	0.99	1/14 (7.1% (0.2 – 33.9%))	1/24 (4.2% (0.1 – 21.1%))	2/38 (5.3% (0.6 – 17.7%))	0.99
80 – 89	0/3 (0% (0 – 70.6%))	2/8 (25% (3.2 – 65.1%))[4]	2/11 (18.2% (2.3 – 51.8%))	0.99	1/3 (33.3% (0.8 – 90.6%))	0/8 (0% (0 – 36.9%))	1/11 (9.1% (0.2 – 41.3%))	0.27
Total	1/28 (3.6% (0.1 – 18.3%))	3/49 (6.1% (1.3 – 16.9%))	4/77 (5.2% (1.4 – 12.8%))	0.99	2/28 (7.2% (0.9 – 23.5%))	2/49 (4.1% (0.5 – 14.0%))	4/77 (5.2% (1.4 – 12.8%))	0.62

1. Fisher's exact test; 2. Colon cancer; 3. Breast Cancer; 4. Breast cancer, Colon cancer + Basal Cell Carcinoma

Supplementary Table 2: One-year prevalence of all cancer sites in Denmark in 2016 (absolute numbers (numbers per 100,000)).

Age groups	Male	Female	Total
50-59	0.6% (494 (570.3))	0.7% (587 (687.0))	0.6% (1081 (628.3))
60-69	1.4% (1065 (1413.0))	1.1% (833 (1089.7))	1.3% (1898 (1250.2))
70-79	2.2% (1209 (2158.2))	1.5% (896 (1477.6))	1.8% (2105 (1804.5))
≥80	2.6% (590 (2552.6))	1.4% (484 (1356.2))	1.8% (1074 (1826.5))
Total	1.4% (3358 (1392.6))	1.1% (2800 (1084.4))	1.2% (6158 (1233.3))
